# Physically real and virtual reality exposed line bisection response patterns: visuospatial attention allocation in virtual reality

**DOI:** 10.3389/fpsyg.2023.1176379

**Published:** 2023-07-24

**Authors:** János Kállai, Tamás Páll, Kristóf Topa, András Norbert Zsidó

**Affiliations:** ^1^Institute of Behavioral Sciences, Medical School, University of Pécs, Pécs, Hungary; ^2^Artistic Research at the University of Applied Arts Vienna, Vienna, Austria; ^3^Institute of Psychology, Faculty of Humanities and Social Sciences, University of Pécs, Pécs, Hungary

**Keywords:** attention allocation, line bisection, neuronal network, pseudoneglect, virtual reality

## Abstract

**Introduction:**

To understand the nature of hemispatial attention allocation in virtual reality (VR), a line bisection task (LBT) was administered both in a real environment and a virtual environment to assess the rate of pseudoneglect. The mental construction of real and virtual environments was assumed to increase visuospatial activity in right hemisphere-related cognitive processes; an alteration in the activity that manifests in the direction and rate of line bisection lateral error.

**Methods:**

In the present study, fifty-one right-handed healthy college students were recruited. They performed a line bisection task in real and virtual environments.

**Results:**

The obtained data showed that LBT errors in real and VR environments were correlated and individually consistent. Furthermore, a leftward LBT error was found in the physically real environment, however, in a VR the line bisection bias drifted towards the right hemispace. Participants with a lower right-handedness score showed a lower rate of left LBT bias in a real environment, but in VR, their LBT error showed a stronger rightwards error.

**Discussion:**

Participants showed an individually consistent pattern in both real and VR environments, but VR-induced visuospatial reality construction was associated with rightward LBT bias in a virtual environment.

## 1. Introduction

Virtual reality (VR) is considered as equipment for developing mental abilities, enhancing knowledge, and facilitating both neurorehabilitation programs in diagnostic and therapy phases of psychiatric and neuropsychological practice (Corbetta and Shulman, 2011; Bisso et al., 2020; Vass et al., 2020; Dellazizzo et al., 2021; Sokołowska, 2021). However, the direct effect of virtual reality on the modification of visuospatial cognition and its interaction with lateralized brain function has not been extensively explored. The somatosensory system defines an individual’s body as a multimodal perceptual and motor system that is localized in a current place and time in physical reality. At the same time, VR users, while immersed in computer-generated VR, are embedded in an alternative visual environment that differs from the physical environment where they are currently used and their perception depends on the technical articulation of the constructed scenes, task demands, and the distance of the presented stimuli from the body (Gamberini et al., 2008; Cléry et al., 2015; Longo et al., 2015).

Performing a task in VR, attention allocation has an enhanced vulnerability in the control of the peri- and extrapersonal taking of visuospatial references. This mentioned cognitive plasticity or instability frequently causes difficulties in users’ recognition of relevant or irrelevant environmental cues and interferes with the integration of somatosensory and visuospatial stimuli (Cardinali et al., 2012). In physical reality, spatial reference taking is focused on peripersonal near, extrapersonal focal, or extrapersonal far space, depending on the spatial distance of the action-guided cues from the body (Rizzolatti and Camarda, 1987; Previc, 1998; Graziano, 2018). The intermediate area of the peripersonal near and extrapersonal focal place is a place, in which the attention allocation dynamically changes, and is considered a mediation zone for searching representation frames for adequate responses (Bufacchi and Iannetti, 2018; Coello and Cartaud, 2021). When forming a mental construction of the space, the change in the distance of the stimuli from the body is associated with activity in the posterior parietal lobe, superior temporal lobe, and decision and action-dependent prefrontal areas. In addition, the different components of visuospatial construction are related to the functional stability of the magno- and parvocellular systems, which are the starting point of the dorsal and ventral attentional allocation stream (Goodale and Milner, 1992). However, the exact nature of perspective-taking in VR is debated. It is currently unclear whether the participant wearing a VR headset uses a peripersonal or an extrapersonal perspective while navigating in a virtual environment. VR is assumed to induce a specific cognitive load to lateral brain functions. In right-handed individuals, the hemispheric lateralization of visuospatial processing is not consistent. Spatial orientation and the construction of peripersonal space require strong right hemispheric activation; however, spatial visualization, visual scanning of an object, feature analysis and definition of an object, and the construction of object-centered space in the focal extrapersonal space demand symmetric hemispheric information loading (Rossetti et al., 1995; Zacks, 2008; Hattemer et al., 2010; Hirnstein et al., 2018). Subsequent analyses need to verify the neuropsychological network that is engaged in this functional duality of the attention allocation and spatial reference-taking systems in VR. In the current study the peripersonal-extrapersonal representation dichotomy has not been investigated, the data-gathering and analyses focused on the behavioral consistency of the line bisection error in physically real and virtual reality environments. The current study uses a line bisection task (LBT) that is appropriate for assessing the direction and rate of hemispatial response bias in healthy participants.

### 1.1. Pseudoneglect and line bisection

Behavioral and neuropsychological studies indicate that the control of visual search does not cover the space’s left and right sides. Studies of healthy individuals (Nash et al., 2010) and patients with hemispatial neglect (Halligan et al., 2003; Kinsbourne, 2006) demonstrated that spatial representation deviation occurs in proprioceptive, haptic, and visuospatial modalities and depends on the respondent’s handedness. Control of visuospatial attention in right-handed individuals leads to the overrepresentation of the left hemispace. The degree of asymmetry in the representation of the visuospatial field depends on whether the involved hemisphere is lesioned, activated, or overactivated; asymmetry can refer not only to the left and right areas of the space but also to differences in attention within those spaces (Fischer, 2001; Foulsham et al., 2013). Furthermore, asymmetry depends on handedness, neurobiological modulators of cognitive control, activation of the dopaminergic system, and variations in several genes that modulate leftward and rightward functional biases in the brain (Benwell et al., 2014; Ocklenburg and Güntürkün, 2018).

The LBT-induced hemispace-specific asymmetric attentional allocation may indicate an asymmetric representation of the space in healthy individuals (Bisiach et al., 1979; Karnath and Rorden, 2012). A large body of evidence has demonstrated that healthy individuals show consistent leftward deviation (pseudoneglect) in the line bisection task (LBT) (Bowers and Heilman, 1980; Fink et al., 2000). The rate of this error from the center of the line depends on handedness, sex, age, and the method of LBT (Jewell and McCourt, 2000; Darling et al., 2012). The attention allocation bias-related representational pseudoneglect measured by LBT and other behavioral observation methods reflects individual differences in the lateralization of visuospatial cognitive function in both human and animal brains (Tomer et al., 2012). Left lateral visuospatial representational neglect is most often associated with right-brain hemispheric lesions but also depends on hemispheric activation, dominance, and the rate of task-dependent activation (Mattingley et al., 2004; Kitadono and Humphreys, 2007; Shulman et al., 2009; Foulsham et al., 2013). The interpretation usually advanced to explain this phenomenon refers to right hemispheric dominance in attentional allocation: Participants overestimate the left side of the space, and thus, their perception of the center of the line drifts leftward. The rate of LBT bias depends on age, sex, handedness, and applied LBT design (Friedrich et al., 2018; Mitchell et al., 2020). Attention allocation to the left side of the hemispace alters the sensitivity of perception in the right hemispace. Right hemispatial perception improved when patients with right hemisphere neglect were exposed to ipsilateral hemispatial visual and haptic synchronous stimulation, but contralateral synchronous haptic and visual stimulation reduced perception. This cross-modal visuospatial extinction points to the fact that cross-modal integration diminishes the perceptual articulation of the space when the right and left hemispaces are synchronously stimulated (Làdavas and Serino, 2008). Competition for attentional resources interferes with multimodal integration by causing mismatches or dissociation in higher-order representations of the body schema in patients with hemispatial neglect as well as healthy individuals. Neuropsychological and fMRI studies with LBT have shown individual neural network variations in pseudoneglect. Nonetheless, the LBT can be considered an adequate measurement for assessing the lateral cerebral basis of spatial attention allocation (Zago et al., 2017; Gerrits et al., 2020).

### 1.2. Hypothesis

The line bisection error is an individually consistent characteristic that may be considered an index of the direction of brain lateralization and attention allocation to the contralateral visuospatial hemispace. In the current study, two hypotheses were tested. *Hypothesis 1:* We predict that the line bisection error can be used as a reliable index of the lateralized attention allocation not only in physical reality but also in virtual realities. *Hypothesis 2:* Since the mental construction of virtual reality influences visuospatial cognitive processing in the right hemisphere, we predict that VR-induced cognitive overload inhibits attention allocation to the contralateral visual field. Consequently, we expect that the rate of leftwards line bisection error will be reduced in virtual reality conditions and drift towards the right hemispace. Namely, the rate of leftward pseudoneglect is expected to be lower in virtual reality conditions than in physical reality.

## 2. Methods

### 2.1. Participants

Fifty-one healthy, right-handed participants – including twenty-four males (mean age = 26, S.D. = 2.1) and twenty-seven females (mean age = 22.5, S.D. = 2.7) – were recruited by public advertisements for participants who were in college or had graduated from college. We have performed a prior analysis for the required sample size regarding the most complex test using the G*Power3 software developed by Faul et al. (2007). In this study, the sample size of 51 individuals satisfies the criteria. Since left- and right-handed individuals exhibit distinct patterns on a visuospatial task (Peters and Servos, 1989), only right-handed individuals were recruited for this investigation. Handedness was assessed by the Edinburgh Handedness Inventory (Oldfield, 1971). The Handedness Laterality Quotient (LQ) was calculated by the following formula LQ = ([Right–Left]/[Right + Left]) x 100. The inclusion criterion for LQ was a score of 70 or higher. No participants had any previous psychiatric or neurologic illness or any prior experience with virtual reality. Before the examination session, all participants underwent acute cognitive and mental state screening, in which general executive functioning was measured by the Trail Making Test parts A and B. All participants’ scores were within the normal range and standard deviations, with no outliers. Participants received a small fee for taking part in the study. This examination is part of a larger study that focuses on exploring the neuropsychological characteristics of the effect of VR induction on brain functions. The sample of the study covers the group of participants that was introduced in a previous examination published by us (Kállai et al., 2022). The investigation was conducted in adherence to the principles of the Declaration of Helsinki and approved by the Regional Research Ethics Committee of the Medical Centre of the local university. Informed consent was obtained from all subjects.

### 2.2. Ethical approval

Ethical Allowance # 2017–2002 146,732 by the Regional Committee of the University of Pécs.

### 2.3. Apparatus

The virtual reality condition was created in the Unity game engine, version 2019.3f1. The application’s target hardware for rendering was a wireless HTC Vive 2018 head-mounted display (HMD) and its controller. The software was written in the C# programming language, and the user interface was created with Unity’s UI kit, which adjusts the interface to proportionally fit all screen sizes. This kit provided a custom user interface designed specifically for laboratory experiments. The software used a hybrid virtual-physical calibration approach to fit the coordinate system of the virtual space in the physical location. On the first screen (the calibration and data input screen), experimenters could calibrate the virtual room with the HTC Vive controllers by entering calibration mode and physically attaching one of the handheld controllers to a cross-shaped target point in the physical room. The physical target point had a virtual double that served as the origin of the virtual space, with the same x-y-z coordinates. The two points were thus synchronized with millimeter-level precision, fixing the two spaces in one coordinate system. This was a crucial step to maximize the precision of measurements collected during the experiment. The calibration data were saved into a JSON file on the local hard drive of the PC that ran the experiment. This calibration file was loaded every time the software was opened. The experimental data, location, length of the lines, and room size were stored locally in JSON files, making them accessible to the laboratory for the creation of new virtual setups and circumstances. The main measurements collected in the experiments were the position, rotation, and relative rotation of the HMD and the triggers. The data collected from these devices were obtained in CVS and stored in .sav files. The data sampling frequency rate of the device was 4 Hz. This program has been applied in several other experiments in our laboratory.

### 2.4. Procedure

The equipment and the technical conditions were identical to a method that we used in a previous study (Kállai et al., 2022) in which the examination focused on the association between the line bisection error and personality characteristics. In the present study, we assessed the effect of VR induction on the lateral hemispatial attention allocation that is tested *via* LBT. Furthermore, the examination aimed to reveal the line bisection error congruency in real and virtual environments. The data gathered come from the same population that participated in a previous examination (Kállai et al., 2022), but the set of variables differed across the two studies. The LBT was presented in focal extrapersonal environments space in both physically real conditions and computer-generated virtual reality. Before VR immersion in a separate room, the LBT baseline was assessed in a physically real environment (RE). The participant stood in front of a screen located 100 cm distance, in the focal extrapersonal space. The LBT consisted of two identical blocks with a 5-s break in between. The design of the LBT was the same in the RE and VR conditions. Participants performed the bisection in the RE condition using a paper screen with the LBT and a 20 cm-long wooden stick was used to perform the line bisection. However, in the VR condition, the wooden stick was replaced by a laser pointer of the VR controller. In both cases, the wooden stick and the laser pointing were visible to the participant. The result of bisection was recorded automatically in the standardized 3D x-y-z coordinate system. The screens where the lines were presented in RE and VR conditions covered 180 degrees of the participant’s visual field. The response sheet involving the lines was positioned in the center of the visual field and the viewing angle of the lines was between 35–45 degrees. The rate of the bisection errors (i.e., bisections that missed the midpoint) in both RE and VR conditions was calculated as a percentage. Leftward errors were indexed as negative bias scores and rightward errors were indexed as positive bias scores.

### 2.5. Measurement

#### 2.5.1. Line bisection task

**Figure 1:**
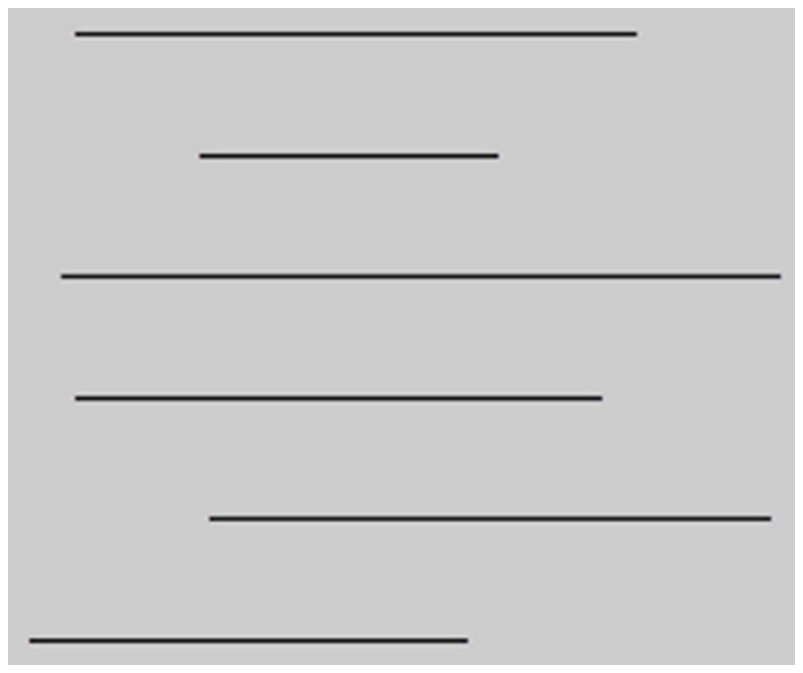
The design of the Line Bisection Task.

Participants were asked to bisect six horizontal lines exactly in their middle with a wooden stick. The six black horizontal lines on the response sheet provided differed in length and location. The width of the lines was standardized (1 mm), but the lines varied from 11 to 27 cm in length. The lines were placed in different positions on a response sheet (see Figure 1). One response sheet contained six lines. After a five sec break, the same response sheet was repeated. Therefore, participants performed 12-line bisections in a short period. Three seconds were allotted for the bisection of each line. Similar methods were reported previously (Egelko et al., 1988; Hausmann et al., 2003; Ocklenburg and Güntürkün, 2018; Kállai et al., 2022). A large body of evidence has confirmed that the line bisection task involves two different attentional mechanisms (person-cantered lateral attention allocation and object-cantered selective attention) and varies according to other personal variables (sex, handedness, test methods, and attention style predispositions) (McIntosh et al., 2017; Mitchell et al., 2020). The line bisection error (LBE) was calculated as the rate of deviation from the true midpoint for the total length of the line and transformed into a percentage. That is, LBE = deviation rate from the middle of the line/true half of the Line x 100.

Negative scores indicate a line bisection bias toward the left hemispace, whereas positive scores indicated a bias toward the right hemispace. The same bisection task was conducted in two conditions. (1) The LBT was applied in a real environment (RE_LBT) and (2) the LBT was carried out in a virtual reality environment (VR_LBT). Participants’ summary scores in the RE_LBT and VR_LBT conditions were the average directional bisection errors. This commonly employed line presentation method (Ocklenburg and Güntürkün, 2018) can eliminate the interpretation issues that originate from the different response biases for line lengths and locations in the presentation of LBT. In the real environment, Cronbach’s α for 12 bisected lines was 0.860. In the virtual reality environment, Cronbach’s α for 12 bisected lines was 0.656.

#### 2.5.2. Handedness

The handedness was assessed by the Edinburgh Handedness Inventory (Oldfield, 1971). The minimum criterion for right-hand dominance was scores of 70% or higher. The handedness mean was 93, with a standard deviation of 7.8 (range: 70–100). There were no significant sex differences in handedness (males *N* = 24, mean (SD) = 93.0 (5.5); females *N* = 27, mean (SD) = 96.6 (4.8); *t* = 1.76, *p* > 0.1).

#### 2.5.3. Computer game activity

Computer games are commonly used in the examined population and may play a role in the existence of current praxis and attitudes towards activities in the digital environment, namely, VR. For this reason, computer game activity, in hours per week, was measured by a self-report scale. Of the participants, 22.2% played computer games for ten or more hours per week, 25.3% played computer games for less than 10 hours per week, and 52.5% did not play computer games. Males played significantly more computer games per week than females (males *N* = 24, mean (SD) = 11.42 (16.4); females *N* = 27, mean (SD) = 1.41 (3.3); *t* = 2.93 *p* = 0.007).

### 2.6. Statistical analyses

First, we tested a version of the line bisection task in physical reality and VR to examine the correlation between the two measures. Both variables were normally distributed (skewness and kurtosis values <|2|; Shapiro–Wilk test of normality *p* > 0.1); thus, we used Pearson correlations. Then, we examined the difference in mean error rates between VR and the real environment using a paired-sample t-test, with Cohen’s d reported as a measure of effect size. Finally, we tested the effects of age, sex, and handedness scores on the LBT error rates in VR and reality with a mixed ANOVA. Spearman correlations were used for age and handedness (as normality was violated based on the kurtosis values: 3.28 and 2.79, respectively), and an independent samples t-test was used for sex differences, as both the normality and homogeneity assumptions were met.

## 3. Results

We tested participants’ line bisection error consistency in RE and VR by correlating the two measures. Both variables were normally distributed (skewness and kurtosis <|2|); thus, we used a Pearson correlation. A strong positive correlation was found between LBT error rates in RE and VR settings (*r* = 0.45, *p* < 0.001, 95% CI = 0.20 to 0.65). Individual correlations varied between *r* = −0.15 and *r* = 0.41. These results indicate that participants’ LBT error scores showed a consistent pattern; persons with higher error scores in a RE showed a higher score in a VR environment, and conversely, smaller error scores in the RE were associated with smaller error scores in the VR.

We then performed a paired samples t-test on LBT error rates between RE and VR. There was a significant difference between the two environments (*t* (50) = 4.68, *p* < 0.001, Cohen’s d = 0.655). Participants showed a strong leftward bias (*M* = −1.62, 95% CI = −2.25 to −0.73) when they completed the LBT in the RE but tended to drift rightward (*M* = 0.35, 95% CI = −0.55 to 1.25) in the VR setting.

**Figure 2:**
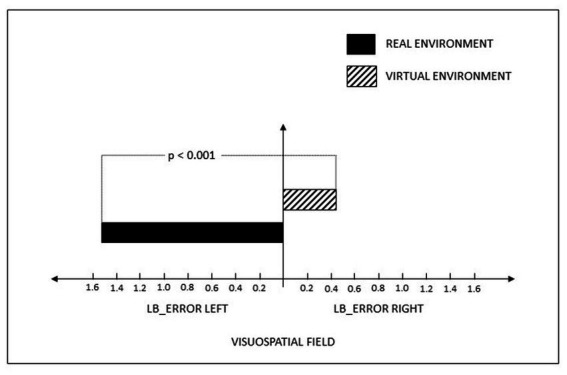
Differences in left and right line bisection errors between the VR and real environments (the error rate from the true midpoint of the lines) as a percentage.

The bisectional error difference from the t-test indicates that in the VR environment, the line bisection error, drifts towards the right hemispace, compared to the error in the RE (see Figure 2). Therefore, attention in the RE is allocated to the left hemispace, and conversely, in a VR environment, the attention is allocated toward the right hemispace.

Participants’ age and computer game experience did not correlate with LBT errors (VR: rho = −0.04, *p* = 0.78 and rho = −0.06, *p* = 0.69; RE: rho = −0.05, *p* = 0.74 and rho = 0.06, *p* = 0.66, respectively). Furthermore, there were no sex differences in the error rates (VR: *t* (49) = 0.66, *p* = 0.51; RE: *t* (49) = 1.42, *p* = 0.16).

**Figure 3:**
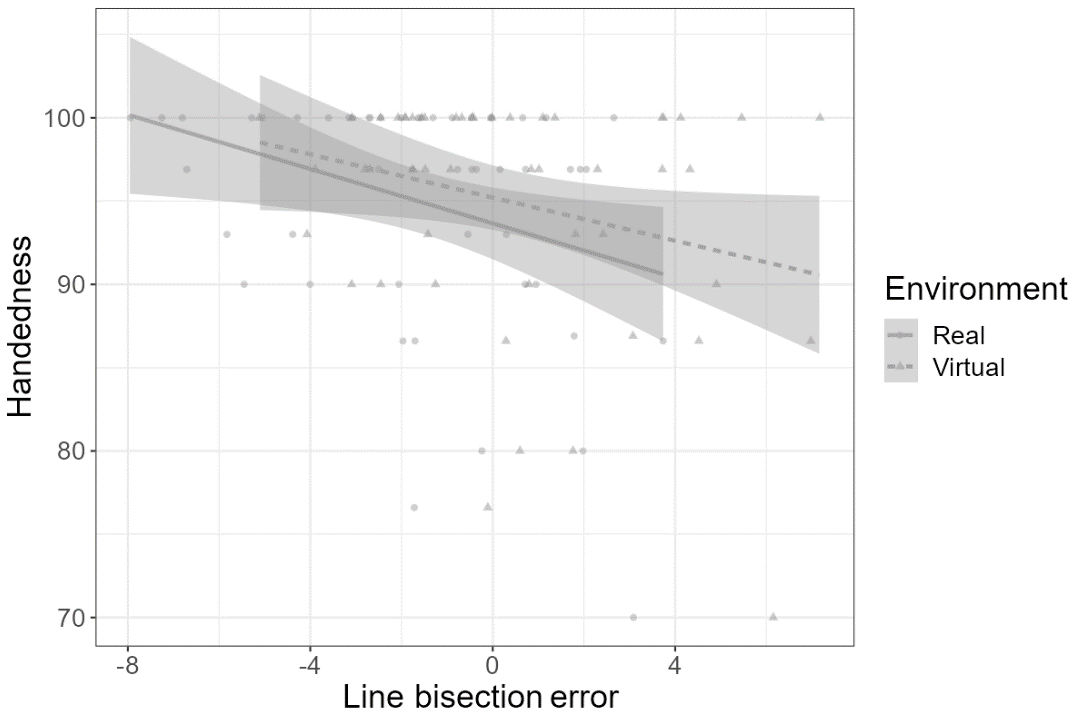
Effect of handedness on the laterality of the line bisection error. Negative scores indicate a deviation from the middle point (0) of the line towards the left side of the Virtual or Real hemispace (in percent of the length of the total lines).

We, then, performed a mixed ANOVA with Environment (LBT errors in RE and VR) as within-subject factors, sex as a within-subject factor, age, and handedness as covariates. Handedness had a significant effect on the LBT error rates (*F* (1,47) = 6.63, *p* = 0.013, ηp2 = 0.124). Follow-up regression analyses (see Figure 3) showed that handedness influenced the magnitude of the LBT error in both the RE (*F* (1,49) = 5.921, *p* = 0.019, β = −0.328) and VR environments (*F* (1,49) = 4.08, *p* = 0.049, β = −0.277). There was no significant main effect of Environment (*F* (1,47) = 0.01, *p* = 0.963), sex (*F* (1,47) = 0.43, *p* = 0.516), and age (*F* (1,47) = 1.93, *p* = 0.171); and no significant Environment x handedness (*F* (1,47) = 0.01, *p* = 0.930), Environment x sex (F (1,47) = 0.36, *p* = 0.549), and Environment x age (*F* (1,47) = 0.06, *p* = 0.801) interaction.

The LBT errors and handedness scores showed a similar regression slope in both the RE and VR environments. Most participants had vivid leftward line bisection errors in the RE (75.55%). However, in the VR condition, the left- and right-line bisection errors were roughly equivalent (left 47%; right 53%), as the bisection error drifted towards the right side of the VR hemispace. Thus, VR alters the rate and the direction of line bisection error in right-handed participants, mainly in cases where the handedness score was low.

## 4. Discussion

This study assessed pseudoneglect in right-handed participants in physically real and computer-generated virtual environments by a line bisection task. Since line bisection errors indicate individually consistent pseudoneglect, we predicted that individuals in both real and virtual environments would show similar response patterns. The obtained results confirmed this expectation. LBT errors in real and virtual environments showed a significant association. Individuals in Real conditions with higher leftward bias showed higher leftward bias in VR conditions, and individuals with lower leftward bias in real conditions showed lower leftward or rightward bias in VR conditions. Therefore, the presented data demonstrated that, despite the consistency in individual LBT errors in Real and VR conditions, most individuals with low leftward dias in real conditions showed a rightward bias in VR conditions. A similar correlating pseudoneglect pattern was found in a study with healthy participants where real and VR-presented cancellation test was used to test for pseudoneglect (Knobel et al., 2020). Therefore, the VR application of the pseudoneglect tests and the LBT among them is a reliable method of assessing the rate of pseudoneglect in healthy participants. The bilateral left or right LBT error pattern is not unique to healthy participants (Revill et al., 2011; Ochando and Zago, 2018). LBT can activate different neural networks to execute the LBT depending on the dominance of the used strategy. Considering the rate of the righthand dominance in our study, participants with lower righthand dominance score tends towards rightward LBT errors. This visuospatial cognitive strategy is thought to be associated with a compensated hemispheric functional laterality and network engagement when individuals allocated attention to bisect a line. Our data align with similar results that have been previously reported (Molenberghs and Sale, 2011; Brooks et al., 2014; Learmonth et al., 2015; Friedrich et al., 2018; Ocklenburg and Güntürkün, 2018). However, our data showed that in the mental construction of a computer-generated virtual environment, in participants with lower-handedness laterality scores, where the laterality rate is compensated, the rightward attention allocation tendency for the visuospatial hemispace is enhanced.

In the interpretation of these results, numerous questions may be raised. Earlier experimental data gained from healthy right-handed individuals (Jewell and McCourt, 2000), it can be statable that visuospatial tasks, visuospatial figurative artworks, and visuospatial exploration activate the right hemisphere, enhance perceptual speed, and accuracy in the left hemispace, and are associated with a leftward bias in the LBT (Ciricugno et al., 2020). Considering the contralateral attention allocation during visuospatial construction, the left hemispace is overrepresented. The functional brain laterality or the brain lesion-related spatial representation bias refers to both the global lateral hemispace and the lateral focal part of the attendant object that is located inside the given hemispace (Fischer, 2001; Foulsham et al., 2013). The line bisection task requires two attentional systems that engage with different cognitive functions that can be conventionally interpreted in the frame of the two visual stream hypotheses (Mishkin and Ungerleider, 1982; Milner and Goodale, 2008; Flindall and Gonzalez, 2017). In right-handed individuals, the LBT induces dorsal stream-related person-centered lateral attention allocation (*“where” pathway*: where a salient stimulus will be present) and ventral stream-related object-centered attentional processes (*“what” pathway*: analysis and recognition of the presented objects) that is the target of action (He et al., 2007; Urbanski et al., 2008; Molenberghs and Sale, 2011; Karnath and Rorden, 2012). The “where” pathway assigns the global place of the potential action, and the “what” pathway identifies the local space of the target and calibrates the target-related action (left-side, right-side, up, down). Therefore, the execution of the LBT involves a dual process in which the procedural and propositional cognitive functions are integrated (Makris et al., 2004; Vossel et al., 2013; McIntosh et al., 2017).

Currently, we have only sporadic experimental data on the integration of “what “and “where” systems in the mental construction of VR. Earlier studies in real environments (Bjoertomt, 2002; Manly et al., 2005; Pérez et al., 2009; Newman et al., 2013) have raised the idea that when an LBT is presented in extrapersonal focal space (viewing distance of about 100 cm) the perceptual load or the length of the test line reduces the attention allocation to the global spatial characteristics and salience of the visual stimuli and that can induce disengagement in ventral attention network that attenuates the left visual field advantage and generates a rightward hemispace bias (Thiebaut de Schotten et al., 2011; Gray et al., 2021). The first represents the physically real environment wherein the individual is currently located (“where” system), and the second represents the visuospatial inputs obtained from the computer-generated VR (“what” system). The VR-immersed participants prefer the visual stimuli of the presented VR (“what”) and partly devalue the representations of the real environment (“where”). This shared attention allocation conflict, the immersion into the real or digital environments, depends on conscious decisions and requires a left hemisphere engagement. Previous data have established that right/left hemispheric compensative activation and elevated conscious control is markedly manifest during VR-induced stimulation (Zacks, 2008).

What conclusions can be drawn from the presented research data? Considering previous results (Weiss, 2000; Hausmann et al., 2003; Corbetta and Shulman, 2011; Thiebaut de Schotten et al., 2011; Mueller et al., 2012), our data support the suggestion that VR may modulate the interhemispheric transfer of information depending on the requirements of the given visuospatial task and the required semantic processing, motoric response, and the selected scanning strategy, which influences the sensitivity of the right and left hemispaces. Previous neuropsychological research (Thiebaut de Schotten et al., 2011; Gray et al., 2021) on the line bisection task has highlighted that interaction among the dorsal and ventral attention networks modulates the role of the semantic process and attention allocation strategy to keep the reality in real and VR environments as well.

The presented results indicate that when a line bisection task is presented in a virtual environment, the line bisection bias in the left hemispace declines and the error drifts towards the right side of VR space. The VR-induced activation of these conflicting cognitive systems modifies the perceptual sensitivity of the left and right hemispaces. Presumably, global attention sensitizes the left hemispace for the intake of stimuli, while focal attention sensitizes the right hemispace for the execution of right-handed actions with defined objects. Since immersion in VR requires a conscious personal decision and induces derealization (active inhibition) of “where” functions for the current physical environment, the left hemispheric attention vector drifts towards the right side of the VR environment. Following (Gray et al., 2021) suggestion for the origin of rightward LBT bias, dual cognitive processing is asymmetrically activated and supports the adaptation to VR and accepts that as a real starting point for goal-directed actions, first of all in participants with lower right-handedness scores.

Another interpretation may be raised for right hemispace LBT error drift in VR. Earlier studies have stated that stimulus distance from the body (near or far) and the visual and haptic-based tool used during performing an LBT induces different neural network engagement in the right parietal lobe originated dorsal visual and ventral stream when an individual construes physically real (Pegna et al., 2001; Holmes et al., 2007; Seraglia et al., 2011; Benwell et al., 2014) or VR environments (Bjoertomt et al., 2009; Cléry et al., 2015). While a healthy individual performs an LBT in a physically real environment and the test line is presented in a relatively near space the right dorsal stream (where-system) that involves the right supramarginal gyrus in the right parietal lobe plays an essential role in the control of attention allocation and action of bisection. The right angular gyrus also contributes to the LBT and the recognition of the lines. The recognition function is associated with the activation of the inferior parietal cortex which is an essential part of the ventral stream (what- system). Therefore, near-space stimuli and response control are shared between dorsal and ventral stream functions, but the activation level of the dorsal system is dominant (Longo and Lourenco, 2010).

Our obtained results are in part supported by the cited studies, but two essential issues can be raised. The first is refer to the type of applied tools (wood stick or laser pointer) to expand the near-space and the second is the location of the stimuli distance from the body that plays an essential role in the result of the bisection (Berti and Frassinetti, 2000; Varnava et al., 2002; Humphreys et al., 2004; Longo and Lourenco, 2010). The result of our examination is consonant with another result where a right hemisphere activation-related left hemispace bias was found during the LBT exposition in a physically real environment (Pegna et al., 2001; Ciricugno et al., 2021). However, the locale content of the stimuli, the changing between visual foreground and background, and the distance from the body boosting the left hemisphere functions may be a role in the rightwards LBE in various presentation conditions (Cardinali et al., 2012; Ciricugno et al., 2021). Considering these suggestions an alternative interpretation of our results can be stated namely, in individuals with low right-hand laterality, the VR-induced decline in the leftwards line bisection bias may be originated from the depletion of processing resources in the right hemisphere. Consequently, the functions of the left hemisphere become predominant and the laterality of LBE becomes compensated or shows a right-ward tendency.

## 5. Conclusion

The present study provides evidence of the feasibility of a VR version of the classical pen-and-paper-based Line Bisection Task. The LBT is a reliable method for measuring pseudoneglect in healthy individuals in VR conditions. This study assessed participants’ tendency to line bisection bias in real and computer-generated virtual environmental conditions. The results suggest that the VR environment modified the line bisection bias and induces a drift towards the right side of the VR hemispace, especially for participants with lower right-handedness scores. The results support the suggestion that VR-induced a highly visualized realistic environment useful digital instrument to define hemispatial preference and pseudoneglect in healthy persons and may be a reliable method in patients during the neuropsychological diagnosis process and neurorehabilitation training as well.

### 5.1. Limitations and outlook

Considering a large population data, the distribution of self-reported hand dominance among males was right 87.4% and left 10.4%; among females 90.1% and left 8.4% (de Kovel et al., 2019). The LBT error bias is differ when the response is performed in dominant or non-dominant hands. This study aimed to assess the LBT error in real and virtual environments only for right-handed individuals. In such a way the presented results and interpretations refer to only right-handed individuals. Further investigation is required for further examinations in this domain. Another open question is the uncertainty around the definition of the peripersonal and extrapersonal space in the virtual environment. Investigations with physically real and VR conditions (Pegna et al., 2001; Longo and Lourenco, 2010) demonstrated that the stimuli distance from the body influences the rate of the LBT error and modifies the symmetric representation of the visual space. Exploring the effect of the VR-induced hemispace preference while an individual performs LBT requires solving two theoretical issues. First, the tool us to perform a line bisection calms a visuospatial and visuotactile integration where the realized bisection is grounded and represented in peripersonal space. Second, the perceived distance of the target object is defined in extrapersonal space. Present research data cannot provide enough evidence to describe the exact nature of the peri- and extrapersonal spatial integration in VR during LBT performing.

## Data availability statement

The original contributions presented in the study are publicly available. This data can be found at the Mendeley Data repository: https://data.mendeley.com/datasets/t8fhzrn5jy/1.

## Ethics statement

The studies involving human participants were reviewed and approved by Ethical Allowance # 2017–2002 146,732 Regional Committee of the University of Pécs. The patients/participants provided their written informed consent to participate in this study.

## Author contributions

JK: conceptualization, methodology, software, supervision, funding acquisition, writing—original draft and reviewing and editing. TP: software, data curation, visualization and writing—original draft. KT: data acquisition, investigation, and writing—original draft. AZ: data analysis, supervision, funding acquisition, writing—original draft and reviewing and editing. All authors contributed to the article and approved the submitted version.

## Funding

This study was supported by the NKFI K-120334 grant (JK); and OTKA PD 137588 and ÚNKP-22-4 New National Excellence Program of the Ministry for Innovation and Technology from the source of the National Research, Development, and Innovation Fund (AZ).

## Conflict of interest

The authors declare that the research was conducted in the absence of any commercial or financial relationships that could be construed as a potential conflict of interest.

## Publisher’s note

All claims expressed in this article are solely those of the authors and do not necessarily represent those of their affiliated organizations, or those of the publisher, the editors and the reviewers. Any product that may be evaluated in this article, or claim that may be made by its manufacturer, is not guaranteed or endorsed by the publisher.
